# An interpretable machine learning model for predicting mortality risk in adult ICU patients with acute respiratory distress syndrome

**DOI:** 10.3389/fmed.2025.1580345

**Published:** 2025-04-25

**Authors:** Wanyi Li, Hangyu Zhou, Yingxue Zou

**Affiliations:** ^1^Tianjin Children’s Hospital (Children’s Hospital of Tianjin University), Tianjin, China; ^2^College of Statistics, Shanxi University of Finance and Economics, Taiyuan, Shanxi, China

**Keywords:** acute respiratory distress syndrome, machine learning, prediction model, mortality, ICU

## Abstract

**Background:**

Acute respiratory distress syndrome (ARDS) is a clinical syndrome triggered by pulmonary or extra-pulmonary factors with high mortality and poor prognosis in the ICU. The aim of this study was to develop an interpretable machine learning predictive model to predict the risk of death in patients with ARDS in the ICU.

**Methods:**

The datasets used in this study were obtained from two independent databases: Medical Information Mart for Intensive Care (MIMIC) IV and eICU Collaborative Research Database (eICU-CRD). This study used eight machine learning algorithms to construct predictive models. Recursive feature elimination with cross-validation is used to screen features, and cross-validation-based Bayesian optimization is used to filter the features used to find the optimal combination of hyperparameters for the model. The Shapley additive explanations (SHAP) method is used to explain the decision-making process of the model.

**Results:**

A total of 5,732 patients with severe ADRS were included in this study for analysis, of which 1,171 patients (20.4%) did not survive. Among the eight models, XGBoost performed the best; AUC-ROC was 0.887 (95% CI: 0.863–0.909) and AUPRC was 0.731 (95% CI: 0.673–0.783).

**Conclusion:**

We developed a machine learning-based model for predicting the risk of death of critically ill ARDS patients in the ICU, and our model can effectively identify high-risk ARDS patients at an early stage, thereby supporting clinical decision-making, facilitating early intervention, and improving patient prognosis.

## 1 Introduction

Acute respiratory distress syndrome (ARDS) is a common clinical syndrome triggered by either pulmonary or extrapulmonary factors and is characterized mainly by persistent hypoxemia accompanied by bilateral infiltrates on chest imaging (1). ARDS has a wide range of causative factors, including infections, noninfectious factors, and systemic inflammatory responses. Among infectious causes, pneumonia is a prevalent infectious cause; among noninfectious causes, pancreatitis, aspiration, severe traumatic shock, and transfusion reactions are frequently encountered (2). The pathophysiological processes of ARDS are highly intricate and involve lung inflammation, endothelial and epithelial injury, increased permeability, and coagulopathy, among other factors (2, 3). Despite more than 50 years of advancements in clinical research on ARDS by clinical experts, no specific drug or medical treatment has been found for curing the disease, and the primary treatment of ARDS has relied predominantly on supportive and conservative therapies (3). Current therapies mainly include protective pulmonary ventilation, prone ventilation, neuromuscular blockade, extracorporeal life support, and glucocorticoids (4). Nonetheless, ARDS remains a predominant cause of elevated mortality in intensive care units (ICUs) (5).

The Berlin definition, proposed in 2012, categorizes patients with ARDS on the basis of the degree of hypoxemia: mild (200 mmHg < PaO_2_/FIO_2_ ≤ 300 mmHg), moderate (100 mmHg < PaO_2_/FIO₂ ≤ 200 mmHg), and severe (PaO_2_/FIO₂ ≤ 100 mmHg) (6, 7). However, the Berlin definition has a limited ability to predict mortality risk in ARDS patients in the ICU. This is particularly applicable to patients with severe ARDS (PaO_2_/FIO_2_ ≤ 100 mmHg), as their unfavorable prognosis significantly affects their quality of life and survival. Severe ARDS is often associated with sequelae such as long-term cognitive deficits, mental health problems, ICU-acquired frailty, pulmonary impairment, imaging abnormalities, and limitations in motor function (8). Past studies have shown that the incidence of ARDS in the ICU is approximately 10% (9, 10), with a mortality rate reaching 40% among ARDS patients in the ICU across more than 50 countries globally (9). Consequently, the development of an effective model to predict the mortality risk of ARDS patients in the ICU is highly valuable for assessing the clinical risk of patients in a timely manner (11).

Artificial intelligence (AI), particularly machine learning, has seen significant advancements and applications in critical care medicine. Previous studies have successfully developed a variety of machine learning models for cancer diagnosis, prognosis assessment, and treatment prediction (12, 13). Furthermore, artificial intelligence and machine learning have demonstrated promise in the early detection, diagnosis, severity assessment, and prognosis prediction of ARDS (1, 14). However, the development and validation of predictive models for the risk of ARDS death in ICU patients are lacking. Owing to the impact of different practice patterns on mortality risk prediction models, continuously updating and optimizing prediction models by existing databases combined with the latest machine learning techniques has become an urgent problem (15). The aim of this study was to develop a death prediction machine model for ARDS patients based on admission data from the ICU of several hospitals, using the most accessible clinical information and laboratory indicators to assess disease severity and predict the risk of death in ARDS patients, to provide decision-making support to clinicians, and to help formulate a more effective treatment plan.

## 2 Methods

### 2.1 Study design

**Figure 1:**
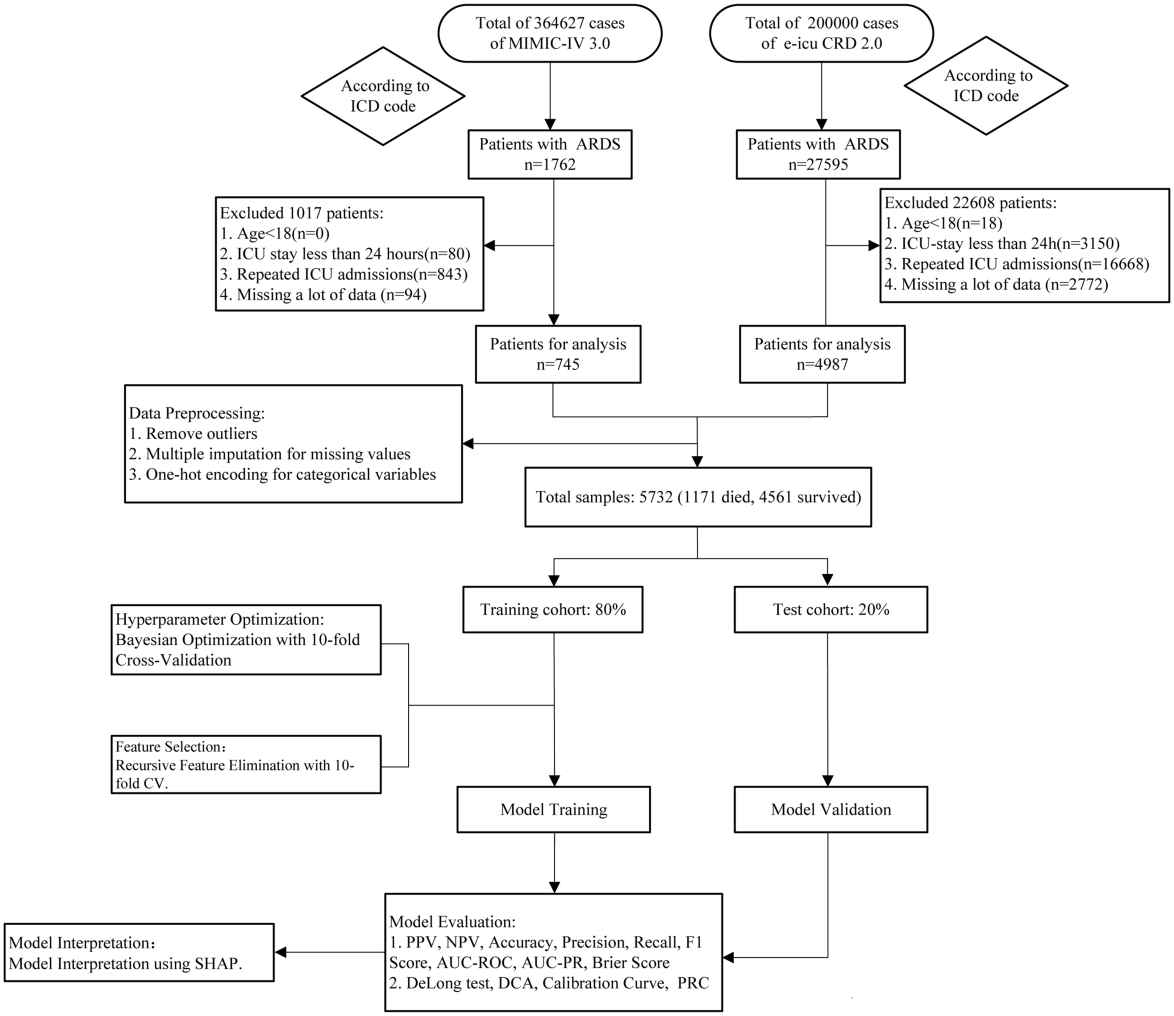
Flowchart of the study design.

This was a retrospective study utilizing data collected on the initial day of ICU admission for ARDS patients, comprising five primary phases: model building, hyperparameter optimization, performance validation, model evaluation, and interpretive interpretation. To guarantee the independence of data assessment, all samples are initially divided at random into a training set and a validation set to prevent any data from leaking between the two datasets. The training set is subsequently used for model development, and the optimal parameter combinations are determined via hyperparameter optimization methods to improve the predictive performance of the model. Next, the model is independently evaluated on the validation set to test its generalizability. To increase the interpretability of the model, this study employed the Shapley additive explanations (SHAP) technique to assess the model and identify the key factors associated with mortality risk in ARDS patients. The overall workflow of this study includes data preprocessing, feature selection, hyperparameter optimization, model training, model evaluation, and model interpretation. The detailed process is illustrated in Figure 1.

### 2.2 Overview of machine learning methods

To construct a prediction model, this study establishes and compares eight machine learning algorithms, including decision tree (DT), gradient boosting tree (GBDT), random forest (RF), LightGBM, XGBoost, AdaBoost, backpropagation neural network (BPNN), and ensemble learning. The DT is used for predicting the output of unknown data via recursive splitting of data, which progressively decomposes the problem into a tree structure for predicting the output of unknown data (16). GBDT significantly improves the prediction accuracy by iteratively training multiple weak classifiers (usually DTs) and optimizing subsequent models on the basis of the prediction error of the previous round (17). RF is also a model that integrates multiple decision trees and reduces the correlation between different decision trees through a bagging mechanism, which effectively reduces the risk of overfitting (18). LightGBM and XGBoost are improved algorithms of GBDT, in which LightGBM adopts histogram-based optimization instead of traditional feature splitting methods, which significantly improves computational efficiency, especially for large datasets; XGBoost further enhances model performance and robustness through gradient optimization, regularization, and weighting strategies (19, 20). The core idea of AdaBoost is to dynamically adjust the sample weights, and in each round of iteration, it increases the weight of the samples that have been incorrectly classified in the previous iteration (21). The BPNN is a multilayer feedforward neural network that adjusts weights and biases via backpropagation and has strong nonlinear modeling capabilities (22). We also integrated multiple models (RF, GBDT, XGBoost, and support vector machine) to construct an ensemble learning model. Ensemble learning usually significantly improves prediction performance and enhances model stability by combining the advantages of different benchmark models. The machine learning models in this study were all implemented in Python (version 3.9.19), built, and trained via the scikit-learn library (version 1.5.1), and the neural network model was developed via PyTorch (version 2.0.0).

The eight machine learning models selected for this study encompass tree-based models, bagging-based methods, boosting-based methods, ensemble models, and neural networks. These models are widely used in clinical prediction tasks, and each has advantages in terms of interpretability, robustness, and computational efficiency, making them suitable for comprehensive comparison in this study (1, 12, 15, 23). In contrast, some other models have relatively weak applicability in clinical prediction tasks. For example, logistic regression may have limited performance when dealing with high-dimensional and complex clinical data (12, 15). The K-nearest neighbor (KNN) algorithm is computationally intensive and is not applicable to large-scale datasets. Deep learning models (e.g., convolutional neural networks) are weakly interpretable, despite their superior performance in certain tasks. In addition, in ICU settings, deep learning models may be at risk of overfitting due to limited sample size. Therefore, when data availability is limited or interpretability is a priority, these models are not the preferred choice. This study prioritizes the use of machine learning models that are mature, stable, and widely used in clinical practice to improve the acceptability and realizability of the models in real-world clinical applications.

### 2.3 Data sources

All clinical data used in this study were obtained from two publicly available independent databases in the United States: The Medical Information Mart for Intensive Care (MIMIC) IV and the eICU Collaborative Research Database (eICU-CRD). MIMIC-IV version 3.0 and eICU-CRD version 2.0 were used in this study. The MIMIC-IV v3.0 database, revised on July 19, 2024, has data from more than 300,000 patients gathered from Beth Israel Deaconess Medical Center between 2008 and 2022 (24). The first author of this study successfully completed the official online course and assessment, thereby obtaining authorized access to the data (Record ID: 13283944). The eICU-CRD includes ICU data from 208 hospitals across the Midwest, Northeast, South, and West regions of the United States, covering over 200,000 ICU patients from 2014 to 2015 (25). Since all the data were deidentified, this study did not require patient consent.

### 2.4 Study population and variables

This study included patients diagnosed with ARDS on the basis of the criteria established by the International Classification of Diseases (ICD-9 and ICD-10). The exclusion criteria included patients under 18 years of age, those who were in the ICU for fewer than 24 h, those with multiple ICU admissions, and those with missing data for more than 20% of the total dataset. Both databases conformed to identical inclusion and exclusion criteria (refer to Figure 1). We used data from the first day of ICU admission for ARDS patients to predict in-hospital mortality and identify potential risk factors. The selected predictors consisted of 6 key categories, encompassing 54 variables that are easy to collect on the first day of ICU admission. These variables included basic information, first-day vital signs, first-day arterial blood gas analysis, laboratory tests, comorbidities, and severity scores.

Specifically, the basic information included age, sex, admission height, admission weight, admission BMI, length of hospital stay before ICU admission, and hours spent in the ICU; first-day vital signs included heart rate, respiratory rate, temperature, systolic blood pressure (SBP), diastolic blood pressure (DBP), mean arterial pressure (MBP), and oxygen saturation (SpO_2_); first-day blood gas analysis included partial pressure of oxygen in arterial blood (PaO_2_), partial pressure of carbon dioxide in arterial blood (PaCO_2_), fraction of inspired oxygen (FiO_2_), PaO_2_/FiO_2_ ratio, and pH; Laboratory tests included hematocrit, hemoglobin, platelet, white blood cell count (WBC), albumin, anion gap, bicarbonate, total bilirubin, blood urea nitrogen (BUN), chloride, creatinine, calcium, sodium, potassium, alanine aminotransferase (ALT), alkaline phosphatase (ALP), and aspartate aminotransferase (AST); and comorbidities included diabetes, renal disease, liver disease, malignant cancer, myocardial infarction, congestive heart failure, peripheral vascular disease, cerebrovascular disease, dementia, chronic pulmonary disease, autoimmune disease, peptic ulcer, and acquired immune deficiency syndrome (AIDS). Additionally, several important scoring systems were included: the Glasgow Coma Scale (GCS), the Acute Physiology and Chronic Health Evaluation (APACHE), the Oxford Acute Severity Index (OASIS), and the Charlson Comorbidity Index (Charlson score).

### 2.5 Data preprocessing

We excluded variables with more than 20% missing data. For variables with less than 20% missingness, we applied multiple imputation by chained equations (MICE) using LightGBM as the imputation model. During the imputation process, MICE generated five imputed datasets, and the final dataset was obtained by averaging these datasets. To ensure that the MICE method did not significantly alter the original data structure, we compared the mean and median differences between the pre-and post-imputation datasets using the bootstrap method and calculated the 95% confidence intervals (CI). The results are provided in the Supplementary material. Differences closer to zero indicate that MICE did not significantly affect the dataset’s mean and median values. The MICE imputation was implemented using the miceforest library (version 6.0.3) in Python. In the dataset used for this study, a degree of class imbalance was observed, with a positive-to-negative sample ratio of approximately 1:4. To improve the model’s ability to identify the minority class, we assigned a higher weight to the underrepresented group (equal to the ratio of the two class sizes) during model training.

### 2.6 Statistical analysis

The patients’ clinical features were delineated as categorical and continuous variables. We eliminated extreme outliers deemed physiologically implausible and removed variables with a missing percentage exceeding 20%. Multiple imputation by chained equations was conducted on the final samples included in the study to address the missing variables (15). Categorical variables were characterized by frequencies and percentages, and the chi-square test was used to compare the differences between the two groups. The distribution of continuous variables was assessed via the Kolmogorov–Smirnov test. Continuous variables that conformed to a normal distribution were described via means and standard deviations (means ± standard deviations), and differences between the two groups of data were compared via independent samples t-tests. Continuous variables that did not follow a normal distribution were described via medians and interquartile ranges, and differences between the groups were assessed via the Mann–Whitney U test. All tests in this study were two-tailed, and the significance level was set at 0.05, with *p* < 0.05 considered statistically significant. The statistical analysis in this study was performed via the SciPy library (version 1.12).

This study thoroughly evaluated the model’s predictive performance via various metrics, including the area under the receiver operating characteristic curve (AUC-ROC), area under the precision-recall curve (AUC-PR), positive predictive value (PPV), negative predictive value (NPV), specificity, recall, accuracy, and Brier score. The prediction threshold was determined by maximizing the F1 score, and all evaluation metrics were calculated on the basis of this threshold. The discriminatory ability of the models was evaluated by AUC-ROC and AUC-PR, and the differences in AUC-ROC between different models were compared using the DeLong test. To mitigate the cumulative impact of the false-positive rate resulting from numerous paired tests, the Holm–Bonferroni correction was applied to adjust the *p*-value of the DeLong test in this study, thus ensuring the reliability and rigor of the statistical conclusions. In addition, the calibration curve is used to assess the consistency between the model’s predicted probability and the actual event occurrence probability; decision curve analysis (DCA) is used to assess the model’s net benefit under different risk thresholds; and the precision-recall curve is used to demonstrate the trade-off relationship between the model’s precision and the recall under different categorization thresholds. The 95% CI for all the evaluation metrics is calculated via the 1,000 bootstrap sampling method to increase the robustness of the results.

### 2.7 Feature selection

Recursive feature elimination (RFE) is mainly used for screening features and identifies and gradually removes features of low importance in an iterative manner until an optimal subset of features is reached (26). Therefore, we use RFE to reduce the dimensionality of the input features and thus reduce the computational complexity. Recursive feature elimination with cross-validation (RFECV) integrates a cross-validation process to assess the model’s performance across varying feature quantities, with the objective of identifying the optimal feature number. This work employed RFE with ten-fold cross-validation to identify the ideal feature subset for models capable of generating feature significance outputs, such as random forest. For models that do not explicitly provide feature importance (e.g., neural networks), the feature subset associated with the model exhibiting optimal final prediction performance was selected.

In this study, RFECV helped to remove the features that were weakly associated with ARDS, improved the stability of the model, and reduced the risk of overfitting. The final selection of features not only improves the computational efficiency of the model but also enhances its interpretability, increasing the model’s practical value in clinical settings.

### 2.8 Model optimization

Bayesian optimization uses a surrogate model (e.g., Gaussian process) to approximate the objective function and finds the optimal solution of the objective function in a defined region through the combination of the surrogate model and the acquisition function (27). The Bayesian optimization updates the prior distribution during iterations to find the optimal hyperparameter combination more efficiently. This method improves the generalization ability of the model while reducing the computational cost, and it makes the final model more robust on the test set. Therefore, we used cross-validation-based Bayesian optimization to obtain the best combination of hyperparameters for machine learning models. K-fold cross-validation partitions the data into several folds, enabling the Bayesian optimizer to assess hyperparameter performance across several data splits, hence mitigating the risk of model overfitting.

This research employed Bayesian optimization with ten-fold cross-validation utilizing the scikit-optimize library (version 0.10.2). Ultimately, all the prediction models were based on the optimal hyperparameter combinations and the filtered optimal feature subsets, and the models were retrained and validated using the training and validation sets to ensure optimal model performance.

### 2.9 Model interpretability

Machine learning models are frequently regarded as “black boxes,” characterized by an opaque and incomprehensible decision-making process. SHAP is a model interpretation method derived from Shapley values that measures the individual contribution of each feature to a machine learning model’s prediction (28). Although some ensemble learning models are able to provide feature importance, these explanations focus mainly on the global level and cannot meet the demand for detailed analysis of individual samples. In contrast, SHAP is not only able to provide global explanations of the model but also local explanations for individual samples with higher accuracy and nuance. Therefore, the SHAP technique is adopted in this study to improve the interpretability of the model and help to understand the role of features in the prediction process in detail.

## 3 Results

### 3.1 Baseline characteristics

A total of 29,357 patients were included in this study, and after 23,625 patients who did not meet the inclusion criteria were excluded, 5,732 patients were ultimately included as the study sample. We categorized these samples into a non-survivor group (*n* = 1,171, 20.4%) and a survivor group (*n* = 4,561, 79.6%). The differences in baseline characteristics between the two groups are shown in Tables 1, 2. Except for the twelve indicators, there were significant differences in all other pathologic characteristics between the two groups. Compared with those in the survivor group, patients in the non-survivor group were older, had a longer length of hospitalization, had higher APACHE, OASIS, and CCI scores, and had lower GCS scores. In the comparison of the first-day vital signs between the two groups, SBP, SpO_2_, PaO_2_/FiO_2_, and pH were lower in the non-survivor group than in the survivor group; in addition, platelets, white blood cells, albumin, anion gap, BUN, bicarbonate, AST, ALP, and ALT were significantly different.

**Table 1:** Baseline characteristics of the study cohort.

Variables	Survival (*n* = 4,561)	Death (*n* = 1,171)	*p*-value
Basic information			
Age(years)	66 (55.0–76.0)	69 (60.0–80.0)	<0.01
Gender (%)			
Male	53	57	
Female	47	43	
Admission height (cm)	168 (160.0–177.0)	168 (162.22–175.3)	0.99
Admission weight (kg)	81.2 (66.0–100.7)	77.6 (64.85–93.65)	<0.01
Admission BMI (kg/m^2^)	28.34 (23.76–34.62)	27.11 (23.06–32.7)	<0.01
Length of hospital stay (d)	8.53 (5.04–15.46)	7 (3.28–13.46)	<0.01
Length of ICU stay (h)	85 (48.0–168.0)	123 (55.96–233.76)	<0.01
First day vital signs			
Heart rate	109 (93.0–126.0)	106 (89.0–123.0)	<0.01
Respiratory rate	32 (23.85–40.0)	29.51 (22.12–36.0)	<0.01
Temperature (°C)	36.56 (36.2–36.9)	36.6 (36.3–36.94)	0.04
SBP (mmHg)	122.2 (102.0–143.0)	107 (76.3–130.0)	<0.01
DBP (mmHg)	71 (60.0–87.0)	72 (58.0–100.72)	0.02
MBP (mmHg)	68 (52.0–119.0)	71.58 (55.0–111.0)	0.43
SpO_2_ (%)	0.96(0.93–0.99)	0.96 (0.93–0.98)	<0.01
First day blood gas			
PaO_2_ (mmHg)	88 (66.8–130.0)	89 (65.15–127.25)	0.60
PaCO_2_ (mmHg)	43.1 (35.5–54.5)	43 (34.45–53.0)	0.01
FiO_2_	0.5 (0.4–0.78)	0.6 (0.4–1.0)	<0.01
PaO_2_/FiO_2_	190 (122.4–286.67)	160 (98.11–255.48)	<0.01
PH	7.36 (7.29–7.42)	7.35 (7.27–7.41)	<0.01
Laboratory parameters			
Hematocrit (%)	34 (29.0–38.85)	32.05 (27.83–37.0)	<0.01
Hemoglobin (g/dL)	11 (9.35–12.75)	10.35 (9.0–12.05)	<0.01
Platelets (K/μL)	400 (292.0–534.0)	318.5 (186.7–474.5)	<0.01
WBC (K/μL)	11.45 (8.28–15.35)	12.3 (8.5–16.86)	<0.01
Albumin (g/dL)	3 (2.6–3.4)	2.7 (2.3–3.12)	<0.01
Anion gap	11 (8.4–14.3)	12.3 (9.0–15.62)	<0.01
Bicarbonate (mmol/L)	25 (21.5–28.5)	23.4 (19.5–27.0)	<0.01
Total bilirubin (mg/d)	0.6 (0.4–0.95)	0.8 (0.5–1.5)	<0.01
BUN (mg/dL)	23 (15.0–37.5)	31 (20.0–47.5)	<0.01
Base excess (mmol/L)	0.4 (−3.2–3.9)	-1 (−6.0–2.3)	<0.01
Chloride (mmol/L)	102.5 (98.5–106.5)	102.5 (98.5–107.0)	0.18
Creatinine (mg/dL)	1.1 (0.79–1.76)	1.35 (0.9–2.16)	<0.01
Calcium (mg/dL)	8.6 (8.1–9.1)	8.4 (7.88–8.9)	<0.01
Sodium (mmol/L)	138 (135.5–141.0)	138 (134.5–142.0)	0.45
Potassium (mmol/L)	4.15 (3.8–4.55)	4.25 (3.85–4.65)	<0.01
ALT (IU/L)	27 (17.0–47.6)	34 (19.0–75.0)	<0.01
ALP (IU/L)	86.5 (66.0–118.0)	95 (69.0–143.0)	<0.01
AST (IU/L)	30 (20.0–52.2)	51 (27.0–122.5)	<0.01
Clinical scores			
GCS	14.82 (12.0–15.0)	14 (10.5–15.0)	<0.01
APACHE	60 (47.0–77.0)	78 (59.0–98.0)	<0.01
OASIS	29 (23.0–37.0)	36 (28.0–43.0)	<0.01
Charlson score	4 (2.0–6.0)	5 (3.0–7.0)	<0.01

**Table 2:** Comorbidities of the study cohort.

Comorbidities	Survival (*n* = 4,561)		Death (*n* = 1,171)		*p*-value
	With (%)	Without (%)	With (%)	Without (%)	
Diabetes (%)	32.58	67.42	30.15	69.85	0.12
Renal disease (%)	16.64	83.36	19.21	80.79	0.04
Liver disease (%)	3.99	96.01	10.42	89.58	<0.01
Malignant cancer (%)	13.81	86.19	21.01	78.99	<0.01
Myocardial infarct (%)	9.73	90.27	11.61	88.39	0.06
Congestive heart failure (%)	22.25	77.75	22.97	77.03	0.63
Peripheral vascular disease (%)	5.37	94.63	6.32	93.68	0.23
Cerebrovascular disease (%)	3.55	96.45	6.58	93.42	<0.01
Dementia (%)	4.65	95.35	5.47	94.53	0.28
Chronic pulmonary disease (%)	27.06	72.94	23.06	76.94	<0.01
Autoimmune disease (%)	2.61	97.39	2.65	97.35	1
Peptic ulcer (%)	2.48	97.52	2.82	97.18	0.58
AIDS (%)	0.3	99.7	0.85	99.15	0.02

### 3.2 Model performance comparison

To ensure the prediction performance of each model, RFECV and Bayesian optimization were applied to each model in this study. RFECV requires the model to be able to output the feature importance to progressively remove features with lower importance in each iteration. However, since the BPNN and ensemble models cannot provide feature importance directly, this study employs the optimal subset of features based on XGBoost (filtered by RFECV) as input features for these models. The features retained by different models after RFECV and their corresponding importance can be viewed in the Supplementary material. In addition, Bayesian optimization combined with ten-fold cross-validation is used to search for the optimal hyperparameter combinations of different models within the specified parameter intervals. The hyperparameter search intervals for all models can be found in the Supplementary material.

We integrated important features from a number of perspectives in our feature screening of key variables, as follows. In the critical care scoring system, APACHE, as one of the important indicators for comprehensively assessing the severity of critical patients’ conditions, integrates multiple information such as physiological parameters, age, and chronic diseases and can effectively reflect the risk of multi-organ dysfunction; Among the blood gas analysis indexes, FiO_2_ and PaO_2_/FiO_2_ are both related to the oxygenation status of patients, which are the core indexes for the diagnosis and severity of ARDS, and low PaO_2_/FiO_2_ is closely related to the destruction of the alveolar-capillary barrier and diffusion dysfunction, and it can be used to predict the respiratory failure and the progression of the disease; Among the laboratory indicators, elevated BUN levels suggest impaired renal function, which is a sensitive marker of the severity of systemic inflammatory response; in addition, although AST is not a specific indicator, its elevation may reflect ischemic injury of hepatocytes or secondary inflammatory factor storm, which suggests the risk of multi-organ involvement.

Among the deleted variables, MBP, as a hemodynamic index, is actively regulated in the early management of ARDS, and its fluctuations are strongly influenced by fluid resuscitation and vasoactive drugs; Mean blood glucose levels were disturbed by insulin therapy, stress, and other factors; Although the anion gap may reflect metabolic acidosis, patients with ARDS are often comorbid with complex acid–base imbalances (e.g., respiratory alkalosis compensation), making it difficult to accurately assess metabolic status, and all of these metrics may weaken predictive stability. Therefore, feature screening ultimately focuses on the most pathophysiologically representative and independently predictive efficacy metrics to improve the clinical interpretability and generalizability of the model.

**Figure 2:**
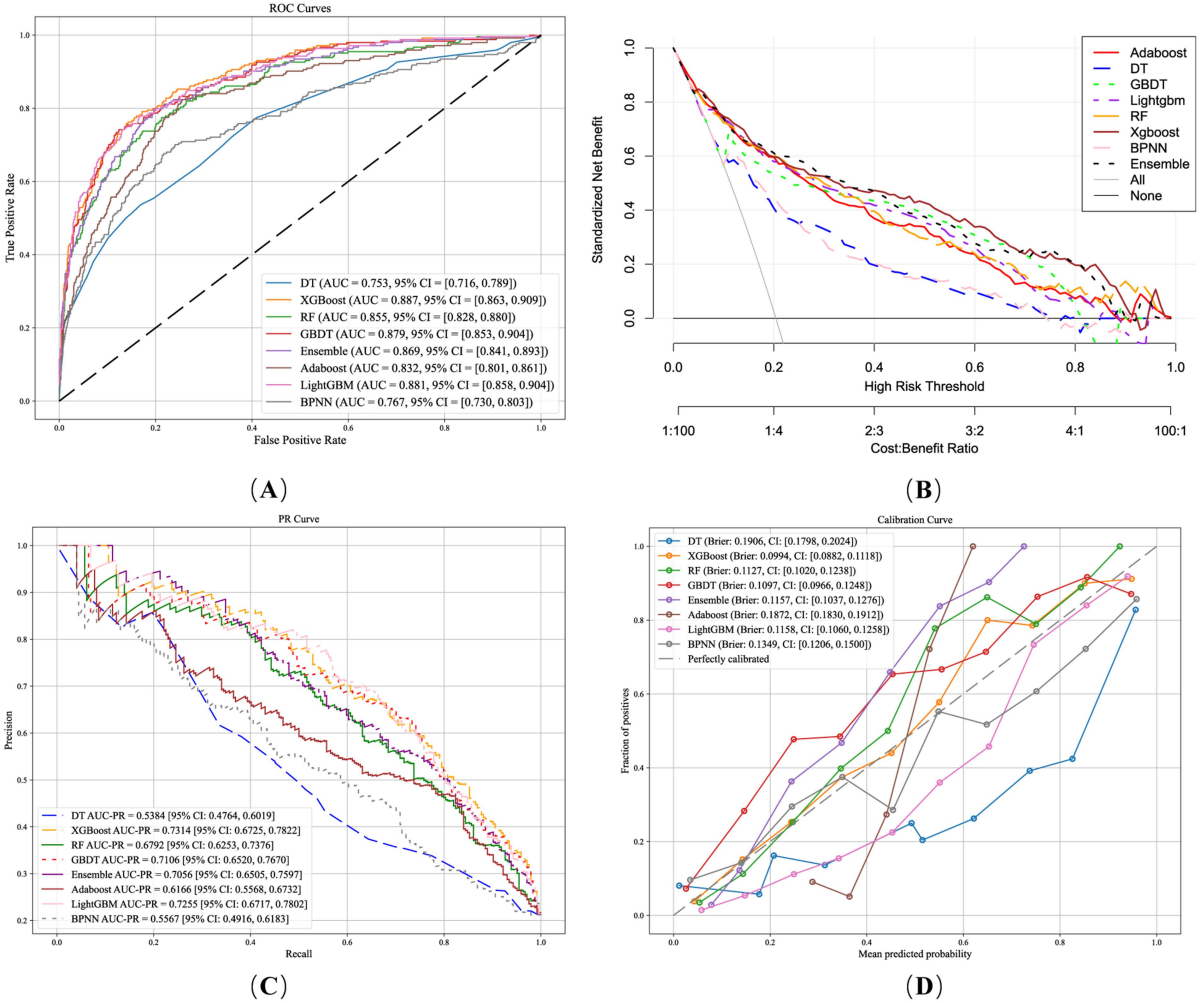
Performance of machine learning models in predicting ARDS mortality. **(A)** Receiver operating characteristic curve. **(B)** Decision curve analysis. **(C)** Precision–recall curve. **(D)** Calibration curve.

After the optimal feature subset and optimal hyperparameter combination are determined, the evaluation metrics of the eight models and their 95% CI are summarized in Table 3. Figure 2A shows the ROC curves of the eight models, and the results indicate that all the models exhibit good discriminatory ability in predicting the risk of ARDS mortality. XGBoost demonstrated excellent performance in terms of AUC-ROC, achieving a value of 0.887 (95% CI: 0.863–0.909). GBDT performed best in terms of NPV, reaching 0.923 (95% CI: 0.904–0.940), and had the highest F1 score of 0.676 (95% CI: 0.625–0.720), outperforming the other models. LightGBM achieved the highest accuracy at 0.846 (95% CI: 0.843–0.882). In contrast, DT, RF, and BPNN showed relatively poorer performance across multiple evaluation metrics. The DeLong test results, after applying the Holm–Bonferroni correction, indicated that the AUC–ROC curve of XGBoost was not significantly different from those of GBDT and LightGBM but was significantly greater than those of the other models. The *p*-values of the DeLong test before and after calibration are detailed in the Supplementary material.

**Table 3:** Evaluation metrics for eight machine learning models to predict the risk of death from ARDS.

Model	AUC	PPV	NPV	Recall	Specificity	F1 Score	Accuracy
DT	0.753	0.537	0.858	0.451	0.895	0.490	0.800
	(0.716, 0.789)	(0.464, 0.603)	(0.834, 0.879)	(0.389, 0.510)	(0.875, 0.916)	(0.435, 0.547)	(0.777, 0.825)
XGBoost	0.887	0.663	0.912	0.676	0.907	0.669	0.858
	(0.863, 0.909)	(0.602, 0.722)	(0.892, 0.930)	(0.619, 0.730)	(0.887, 0.925)	(0.618, 0.716)	(0.837, 0.877)
RF	0.855	0.649	0.894	0.598	0.913	0.623	0.846
	(0.828, 0.880)	(0.585, 0.707)	(0.872, 0.913)	(0.542, 0.656)	(0.894, 0.931)	(0.571, 0.668)	(0.826, 0.866)
GBDT	0.879	0.632	0.923	0.725	0.886	0.676	0.852
	(0.853, 0.904)	(0.576, 0.684)	(0.904, 0.940)	(0.671, 0.782)	(0.866, 0.907)	(0.625, 0.720)	(0.831, 0.873)
Ensemble	0.869	0.610	0.908	0.668	0.885	0.638	0.839
	(0.841, 0.893)	(0.552, 0.665)	(0.888, 0.927)	(0.609, 0.727)	(0.864, 0.906)	(0.588, 0.685)	(0.816, 0.859)
AdaBoost	0.832	0.487	0.926	0.770	0.781	0.597	0.779
	(0.801, 0.861)	(0.439, 0.538)	(0.908, 0.945)	(0.718, 0.821)	(0.754, 0.806)	(0.551, 0.638)	(0.754, 0.802)
LightGBM	0.881	0.688	0.909	0.660	0.919	0.674	0.864
	(0.858, 0.904)	(0.630, 0.744)	(0.891, 0.929)	(0.601, 0.721)	(0.903, 0.937)	(0.621, 0.718)	(0.843, 0.882)
BPNN	0.767	0.487	0.888	0.615	0.825	0.543	0.780
	(0.730, 0.803)	(0.432, 0.544)	(0.865, 0.908)	(0.561, 0.674)	(0.801, 0.849)	(0.489, 0.592)	(0.757, 0.804)

To further analyze the clinical application value of different models in predicting the risk of mortality in ARDS patients, DCA curves were plotted in this study (Figure 2B). The DCA curves revealed that the net benefit of the XGBoost model was greater than that of the GBDT model and the LightGBM model over risk thresholds ranging from 0 to 1. This suggests that under several different decision thresholds, the prediction results of XGBoost are capable of more effectively assisting in clinical decision-making by increasing the identification of true positive cases while reducing the occurrence of false positive cases. In addition, Figure 2C shows the precision-recall curve and calculates AUC-PR. Owing to the severe imbalance between positive and negative samples in ARDS-related data, the traditional ROC curve may exhibit an overly optimistic tendency, whereas the PR curve is more sensitive to the predictive performance of positive class samples; thus, the AUC-PR is more suitable for measuring the predictive ability of the model in positive class samples. The AUC-PR of XGBoost is 0.731 (95% CI: 0.673–0.783), which is higher than those of the other models. Figure 2D shows the calibration curve of the model, which is used to assess whether the predicted probabilities output by the model can accurately reflect the actual probability of an event occurring. The closer the calibration curve is to the diagonal line, the closer the predicted probability of the model is to the real occurrence probability. The Brier score is used to measure the deviation between the predicted probability of the model and the actual result, and the smaller the Brier score is, the better the calibration performance of the model. In this study, XGBoost has the lowest Brier score of 0.099 (95% confidence interval: 0.088–0.112), which is better than those of the other models. In summary, XGBoost performed well on several evaluation metrics, especially compared to other models, in terms of discriminatory ability, calibration performance, and prediction ability for positive class samples. Therefore, XGBoost was identified as the model of choice for predicting the risk of mortality from ARDS in this study.

### 3.3 Subgroup analysis

**Figure 3:**
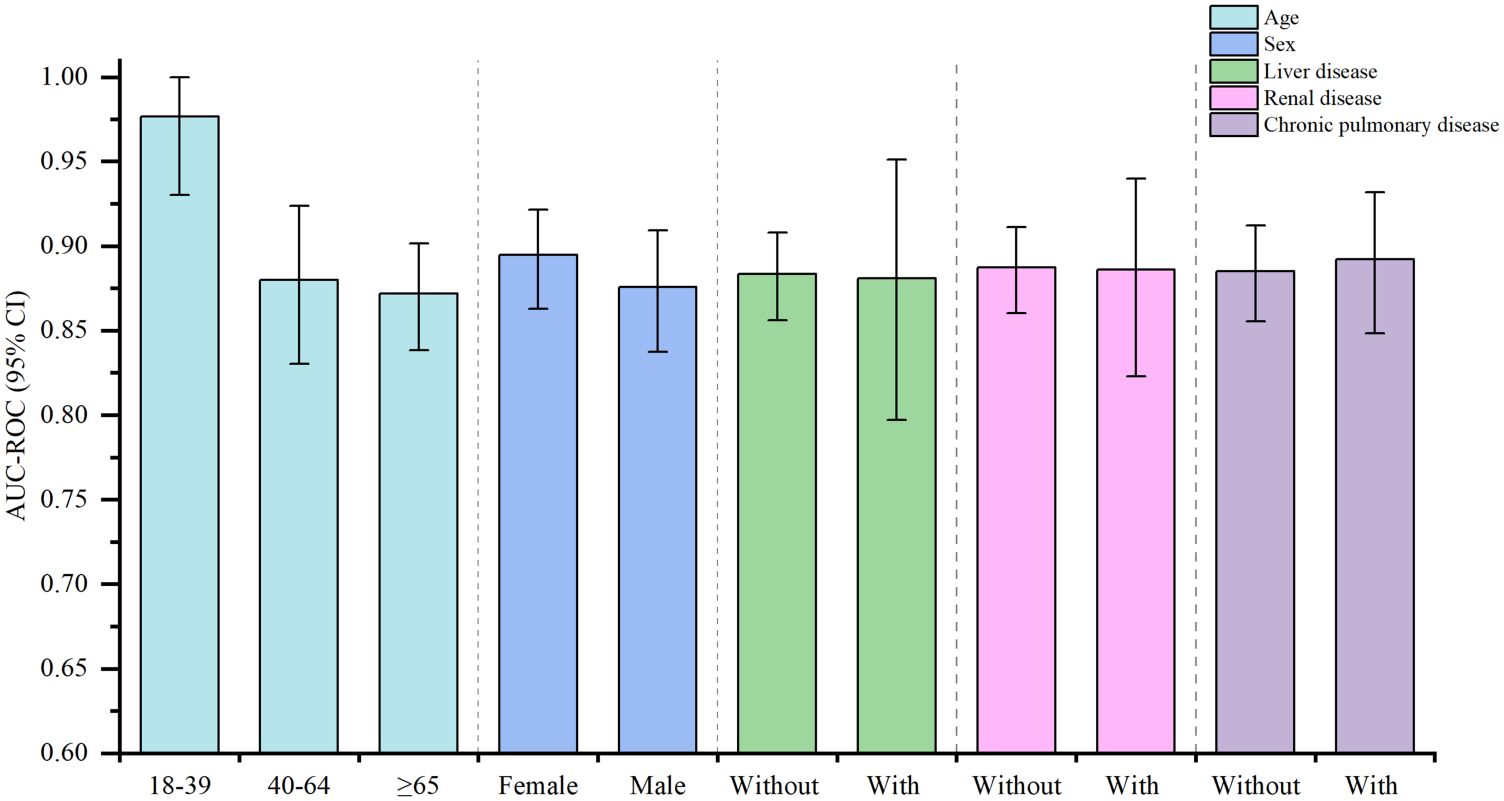
Subgroup analysis of XGBoost for predicting risk of mortality in patients with ARDS.

To further evaluate the performance of XGBoost in predicting mortality risk in ARDS patients, we performed five types of subgroup analyses based on age, sex, liver disease, renal disease, and chronic pulmonary disease. Detailed results of the subgroup analyses are presented in the Supplementary material and Figure 3 illustrates the bar chart of the subgroup analyses. XGBoost demonstrated high discriminatory power across age groups, with the highest AUC-ROC of 0.976 (95% CI: 0.93–1) in the 18–39 years age group, while its predictive performance was comparable between the 40–64 age group (AUC-ROC = 0.88, 95% CI: 0.830–0.924) and the ≥ 65 age group (AUC-ROC = 0.872, 95% CI: 0.838–0.901). Sex-based analysis indicated that the model performed slightly better in male patients (AUC-ROC = 0.895, 95% CI: 0.863–0.922) than in female patients (AUC-ROC = 0.876, 95% CI: 0.837–0.909).

In the comorbidity subgroup analysis, XGBoost demonstrated stable predictive ability across different comorbidity categories. Specifically, in patients with liver disease, the AUC-ROC of the model was 0.88 (95% CI: 0.797–0.951), which was slightly lower compared to the other comorbidity groups, whereas it demonstrated higher predictive accuracy in patients with chronic pulmonary disease (AUC-ROC = 0.892, 95% CI: 0.848–0.931).

These findings suggest that XGBoost maintains strong discriminatory ability in predicting ARDS mortality risk across diverse patient populations, highlighting its potential clinical applicability.

### 3.4 Interpretability

Understanding how machine learning models make decisions on the basis of different features is crucial for researchers and clinicians. Therefore, this study combines the feature importance of the XGBoost output and SHAP to enhance the interpretability of the model. XGBoost’s feature importance primarily provides a global explanation, measuring the significance of each feature in the overall model decision-making process. In contrast, the SHAP method not only offers a global explanation but also provides more detailed local explanations for individual samples.

**Figure 4:**
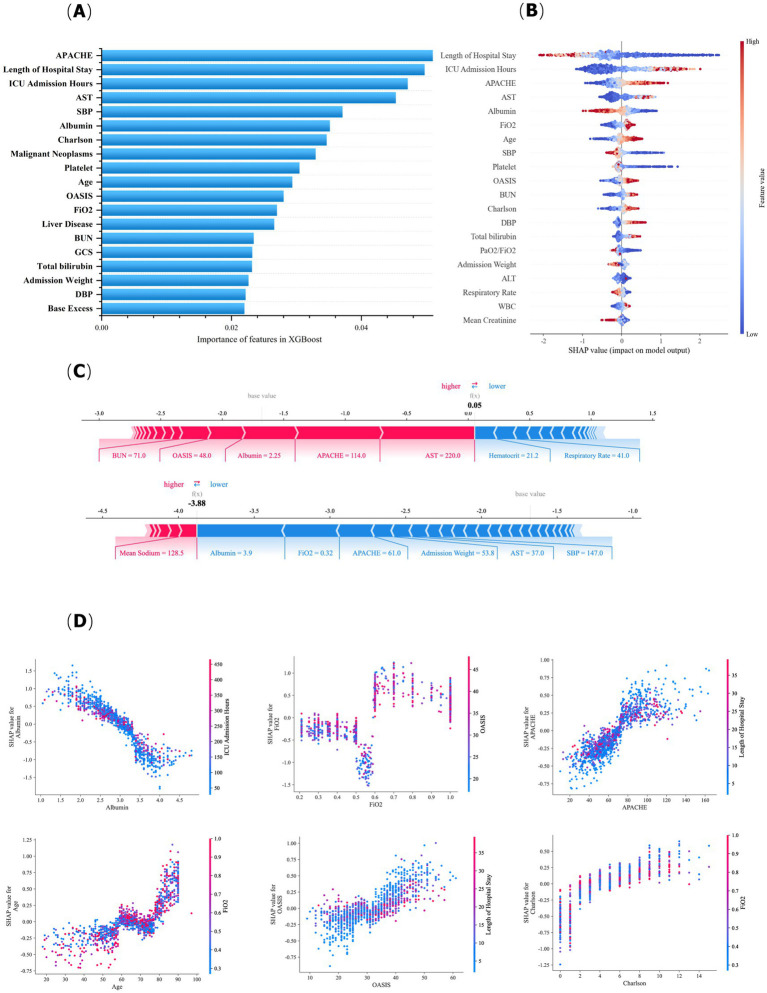
Explanation of ARDS mortality risk prediction models. **(A)** Top 20 risk factors predicted by XGBoost. **(B)** Top 20 risk factors for the SHAP. **(C)** A nonsurviving patient and a surviving patient. **(D)** SHAP dependence plot.

Figure 4A illustrates the ranking of feature importance derived from the XGBoost model output. The principal features include APACHE, length of hospital stay before ICU admission, length of ICU stay, AST, SBP, albumin, Charlson, history of malignant cancer, platelet, age, OASIS, FiO_2_, history of liver disease, BUN, GCS, total bilirubin, admission weight, DBP, and base excess.

Figure 4B shows the SHAP summary plot of the XGBoost model, which provides enhanced insights. For example, when a patient has elevated AST levels, the associated SHAP value is positive, indicating that APACHE is a significant contributor to increased mortality risk; furthermore, as APACHE levels increase, patient mortality risk increases accordingly. The SHAP force plot for two patients is shown in Figure 4C. The upper plot shows the force plot for a non-surviving patient, and the bottom shows the force plot for a surviving patient. For non-survivors, the model predicts a high mortality risk, primarily due to low albumin, elevated APACHE, high AST, and BUN, all of which collectively increase the mortality risk.

Figure 4D illustrates the impact of alterations in individual feature values on SHAP values and examines potential relationships among features. Using FiO_2_ as an illustration, when FiO_2_ levels were maintained between 0.2 and 0.5, the corresponding SHAP values ranged from-0.5 to 0, indicating that, at this stage, FiO_2_ negatively influenced the model’s ability to predict decreased mortality risk from ARDS, suggesting that the patient’s condition was more stable and had not yet progressed to severe respiratory failure in this range of oxygen support. As FiO_2_ increased to between 0.5 and 0.6, the SHAP values further decreased, indicating that an increase in FiO_2_ had a more severe negative impact on mortality risk within this range, implying that the patient received increased oxygen support at this stage. When FiO_2_ surpassed 0.6, the SHAP values were positive, indicating that an increase in FiO_2_ at this stage increased the risk of mortality. This phenomenon indicates that the patient may have reached a critical condition necessitating mechanical ventilation or extracorporeal membrane oxygenation, resulting in a higher risk of mortality. The Supplementary material, further illustrates SHAP’s interpretation of XGBoost in predicting mortality risk in ARDS patients.

## 4 Discussion

In this retrospective study, we developed, optimized, validated, evaluated, and interpreted a mortality prediction model for ARDS patients aimed at predicting the severity and mortality of patients on the basis of their clinical information on the first day of ICU admission. We constructed eight machine learning models on the basis of 54 physiological variables and found XGBoost to be the best performer on the basis of model performance comparisons. Using characteristic importance analysis, we identified the 20 clinical variables that have the greatest impact on ARDS patients, in order of importance: AST, length of hospital stay, SBP, APACHE, ICU admission hours, FiO_2_, total bilirubin, platelets, OASIS, Charlson score, albumin, ALT, malignant neoplasms, age, BUN, ALP, DBP, SpO_2_, PaO_2_/FiO_2_, and PaO_2_. Most of these variables are readily available at the time of the patient’s admission to the ICU; thus, the predictive model we have developed can provide clinicians with a prediction of mortality risk on the first day in the ICU, helping to make more timely and accurate clinical decisions.

To date, a number of researchers have worked on developing machine learning predictive models of incidence and mortality in ARDS patients, for example, Maddali et al. developed machine learning models for identifying ARDS subphenotypes, with AUC-ROCs of 0.92 (95% CI: 0.90–0.95) and 0.88 (0.84–0.91) for their EARLI and VALID clinical classification models, respectively, (29); Lin et al. used XGBoost to build a machine learning model for predicting deaths from non-sepsis ARDS, which predicted NPS-ARDS with an accuracy of 78.0% in external validation (1), Huang et al. developed a prediction model for in-hospital mortality in patients with ARDS using the RF model, and the RF model predicted in-hospital, 30-day, and 1-year mortality rates with AUC-ROCs of 0.891, 0.883, and 0.892, respectively, and showed good generalization ability in cross-dataset validation (23); The XGBoost hyperparametric optimization model constructed by Patel et al. using the blood gas analysis metric SpO_2_/FiO_2_ also achieved good results, with an AUC-ROC of 0.85 on the test set (30).

Compared with existing ARDS prediction models and traditional clinical scoring systems, the interpretable machine learning model developed in this study demonstrated unique advantages; we overcame the limitation of heterogeneity of single-center data through a multicenter cohort design, improved the generalizability of the model in diverse ICU populations, and integrated a number of clinical indicators of the patients (covering vital signs, laboratory tests, comorbidities, organ function scores, and other multidimensional data), which compensated for the lack of coverage dimensions of physiologic indicators in previous studies; In addition, the combination of the Bayesian optimization algorithm and the recursive feature elimination technique has some methodological improvements compared with previous studies on ARDS predictive models, and we constructed a more efficient and accurate subset of features with trade-off properties, and the final XGBoost predictive model established in this study demonstrated a good predictive efficacy on the test set (AUC-ROC: 0.887, 95% CI: 0.863–0.909), and its prediction performance is better than the traditional criticality scoring system (31). More importantly, the clinical value of this study is reflected in the fact that our study improves the interpretability mechanism under the premise of guaranteeing the prediction accuracy. By introducing the SHAP value, we have solved the limitations of the previous “black-box” machine-learning models in the field of intensive care and provided clinically actionable interpretations, which further enhance the practicality of the application of the model to ICUs and clinicians’ applications. This combination of predictive performance and interpretability represents an advancement in the AI tools available for clinical work. As the application of machine learning models in healthcare continues to deepen, it will usher in a new era of healthcare management systems (32).

The “black box” nature of machine learning refers to the fact that its decision-making process is typically not understandable to users. Although these models usually demonstrate high accuracy and predictive power for tasks, clinicians rely on clear reasons and evidence to make decisions. Therefore, improving the interpretability of machine learning models is key to their widespread application in the medical field. This work employed SHAP to elucidate and investigate the decision-making process of machine learning models in predicting the mortality risk associated with ARDS. SHAP aims to provide interpretability for black-box models, making the impact of each feature on the model’s predictions transparent and understandable. SHAP not only explains individual predictions, showing which specific features positively or negatively influence the prediction, but also aggregates the contributions of all data points to provide the overall feature importance of the model, thus helping us understand the model’s general behavior. This study also presents local sample explanations for both non-surviving and surviving patients, further enhancing the interpretability of the model.

Our findings align with expectations, indicating that the total length of hospital stay and duration of ICU admission are critical indicators of disease severity and prognosis. Previous studies have demonstrated that the length of hospitalization in ARDS patients is highly correlated with the severity of the disease (33). Moreover, the mortality rate of critically ill patients in the early stage of deterioration can be significantly reduced if they are admitted to the ICU in a timely manner to receive interventional treatments such as respiratory support or fluid support. Conversely, postponed ICU admission is positively correlated with in-hospital mortality (34, 35). The period of ICU admission accurately indicates the onset of increased therapy following the deterioration of the patient’s health, and as a time-dependent dynamic indicator, it can be combined with condition scores (e.g., APACHE, OASIS) in the machine-learning model of this study to further enhance the evaluation of patient severity and mortality risk. The length of hospital stay may indicate that the patient has undergone a complex course of treatment accompanied by multiple complications, such as secondary infections, sepsis, or multiple organ dysfunction, which aggravate the condition of patients. However, a longer hospitalization may also mean that the patient is likely to eventually recover after a prolonged course of treatment. In the predictive model, the length of hospital stay can be combined with disease scores (e.g., Charlson score) in a stratified analysis to better explain the impact of length of hospitalization on mortality risk.

This study revealed indices of liver function impairment, including AST, total bilirubin, and ALT, as important determinants of severity and mortality risk in ARDS patients, with AST being the most influential according to the XGBoost model. Elevated AST, ALT, and total bilirubin levels typically indicate liver impairment. Compromised liver function results in a diminished capacity to eliminate inflammatory factors, toxins, and metabolic waste, which in turn exacerbates the systemic inflammatory response and further worsens the condition of patients with ARDS. Liver impairment in patients with ARDS is frequently attributed to hypoxia, the systemic inflammatory response, or sepsis, which are key pathological mechanisms in the progression of ARDS (36). Increased AST levels may also indicate the emergence of multiple organ dysfunction syndrome (MODS). Previous studies have shown that elevated AST levels are significantly associated with a decreased PaO_2_/FiO_2_ in ARDS patients, and when AST levels are significantly elevated, patient mortality risk increases substantially (37). Therefore, AST levels can be used as important markers to reflect the state of liver function and the existence of MODS. In patients with ARDS, which is often accompanied by a systemic inflammatory response, hypoxia, and multiorgan failure, especially in patients with impaired liver function, elevated AST, ALT, and total bilirubin suggest the potential for liver failure. Likewise, indicators of impaired renal function, such as BUN, were also included as risk factors in this study. An elevated BUN level suggests that patients may have fluid retention and metabolic waste accumulation, serving as crucial indicators of renal function and protein metabolism in the body. Some studies have demonstrated that in ARDS patients, elevated BUN levels suggest renal impairment, which is especially common in critically ill patients, and high BUN levels significantly increase the risk of death in ARDS patients (38). Acute kidney injury (AKI) is a prevalent consequence in individuals with ARDS (39). These abnormal indicators imply that MODS may significantly contribute to mortality in severe ARDS patients, in which liver and kidney failure are usually the earliest organ failures manifested in MODS and play a crucial role in the prognosis of ARDS patients.

This study highlights the importance of three grading systems for the early identification of high-risk severe ARDS patients: APACHE, OASIS, and Charlson score. The APACHE scoring system is a widely utilized tool for predicting the mortality of patients in the ICU. It amalgamates several physiological indications (including blood pressure, oxygenation index, body temperature, etc.) as well as the patient’s chronic health status to provide a comprehensive assessment of disease severity. A higher APACHE score indicates that the patient’s condition is more critical and may be accompanied by organ dysfunction or a systemic inflammatory response, with a greater risk of death (40, 41). The APACHE scoring system is extensively utilized in ICU mortality risk prediction and is equally effective in severe ARDS patients. Studies have shown that for ARDS patients, the APACHE score is an important tool for the early identification of high-risk patients, with higher scores indicating greater mortality risk (42). The dynamic monitoring of APACHE scores in ICU patients helps clinicians track the effectiveness of treatments. A decrease in the APACHE score as treatment progresses suggests improvement, whereas persistently high scores indicate poor prognosis. Compared with the APACHE score, the OASIS score is more straightforward and user-friendly to apply. The OASIS score integrates physiological indicators (blood pressure, respiratory rate, etc.) and underlying disease information. Consistent with the APACHE score, the higher the OASIS score is, the worse the prognosis. Research on ICU patients indicated a strong correlation between the OASIS score and death in ARDS patients, especially in those with an OASIS score greater than 40, where the risk of mortality markedly escalates (43). In addition, the CCI is a tool that scores patients on the basis of their underlying comorbidities, such as heart disease, diabetes mellitus, liver disease, kidney disease, and malignancy. In this study, an elevated CCI score was strongly associated with an increased risk of death in patients (44). In patients with ADRS, an increase in the CCI score indicates a higher burden of underlying disease and a higher risk of death, especially in elderly patients, where the impact of the CCI score on patient prognosis is more pronounced.

FiO_2_ reflects the patient’s oxygen requirements, and higher FiO_2_ values generally signify that the patient requires increased oxygen to sustain arterial oxygen equilibrium, which at the same time may suggest that the patient’s lung function is severely impaired, indicating the severity of ARDS. Numerous studies have shown that FiO_2_ is inversely linked with the PaO_2_/FiO_2_, suggesting that elevated FiO_2_ is correlated with poorer prognoses for ARDS patients (45). Furthermore, extended exposure to high FiO_2_ may also increase mortality risk in critically sick patients, probably because prolonged use of high oxygen concentrations (FiO_2_ > 60%) leads to oxygen toxicity, worsening lung tissue damage and perpetuating a detrimental cycle. Therefore, FiO_2_ serves as a critical indicator for assessing disease severity and determining oxygen support strategies in patients with ARDS. When treating critically ill ARDS patients in the ICU, FiO_2_ should be dynamically adjusted according to the patient’s oxygenation status to maintain appropriate oxygenation levels. The significance of FiO_2_ is particularly apparent in mortality prediction models, and when used in combination with PaCO_2_, SBP, and other indicators, it can yield a more thorough assessment of a patient’s respiratory and circulatory function.

Moreover, other additional indicators deserve attention. Many studies have shown that the albumin concentration is a significant independent risk factor for the progression of ARDS (46). Researchers have reported that the lactate–albumin ratio (LAR) serves as a reliable predictor of risk in acute respiratory distress syndrome patients and is positively correlated with 28-day mortality in ARDS patients (47). Our study further confirmed the significance of the albumin concentration. The fundamental pathophysiological characteristic of ARDS is the heightened permeability of the alveolar-capillary barrier, and hypoalbuminemia is a typical manifestation of this pathological state, which may exacerbate the occurrence of non-cardiogenic pulmonary edema. In addition, lower albumin decreases colloid osmotic pressure, further exacerbating body fluid leakage. It has been shown that body fluid overload in ARDS patients is closely related to the length of hospital stay, duration of mechanical ventilation, mortality rates, and overall prognosis (48). Therefore, albumin concentration directly impacts a patient’s fluid balance, thus indirectly influencing their prognosis. These findings suggest that albumin replacement therapy may be a potential method for enhancing pulmonary symptoms in patients with ARDS.

Age has historically been regarded as an important risk factor for ARDS patients (49), which aligns with the results of this study’s predictive model. Studies have demonstrated that age is an important independent predictor of mortality in ARDS patients, particularly for those aged 65 and older, whose mortality rate is significantly higher than that of younger patients (50). With advancing age, patients experience a steady decline in organ reserve and compensating capacity. Geriatric individuals frequently present with multiple chronic conditions, including cardiovascular disease, diabetes, chronic obstructive pulmonary disease (COPD), and malignancies, and typically exhibit higher CCI scores. The combination of these factors results in a markedly elevated mortality risk for older patients with ARDS.

Surprisingly, arterial blood gas analysis indicators, including SpO_2_, PaO_2_/FiO_2_, and PaO_2_, were ranked last in the mortality prediction model of this study on the initial day of ICU admission. Although low SpO_2_, low PaO_2_, and low PaO_2_/FiO_2_ are fundamental clinical manifestations of ARDS, evidence suggests that the PaO_2_/FiO_2_ classification in the Berlin definition has not been confirmed as a predictive factor for mortality in ARDS patients (11, 51). This result indicates that the mortality rate of ARDS patients is not always directly correlated with the severity of their condition; mortality is also further influenced by various other factors. Similarly, the prognosis of ARDS patients does not depend solely on lung-related indicators. However, since we only collected vital signs and arterial blood gas analysis data from the first day of ICU admission and did not capture the trend of changes in SpO_2_, PaO_2_/FiO_2_, and PaO_2_ during the treatment process, this may be a key reason for these findings. Therefore, clinicians should still consider a combination of SpO_2_, PaO_2_/FiO_2_, and PaO_2_ holistically when making decisions.

In the ICU, the construction of accurate and interpretable prediction models is essential for treatment decisions and resource allocation for patients with ARDS. Especially when clinical decisions need to be made quickly, the early warning provided by the model can help medical staff to intervene and manage in a targeted manner with limited time and resources. The SHAP method demonstrates certain application values in clinical practice. First, SHAP values can reveal the characteristics that contribute most to the risk of death in ARDS patients, thus providing a basis for the development of individualized treatment strategies. Secondly, by dynamically observing the trend of SHAP values, clinicians are able to detect the risk and intervene promptly when the patient’s condition changes slightly. In addition, the SHAP method helps to assist in diagnostic and therapeutic decision-making: for high-risk patients, active interventions can be taken based on key indicators, such as adjusting ventilation strategies or increasing monitoring frequency, while for low-risk patients, interventions can be appropriately reduced, thus optimizing resource allocation. Combined with the results of SHAP analysis and clinical monitoring indicators, medical staff can better balance the risks and benefits of therapeutic interventions and ultimately improve the prognosis of patients and the efficiency of resource use.

However, there are still some limitations to the interpretive nature of SHAP in complex and severe clinical situations. First, the SHAP value mainly reflects the contribution of local changes in the model to the predicted results, and there are often complex interactions between multiple variables in clinical data, and it may be difficult for a single SHAP analysis to fully capture these effects. In addition, SHAP provides model-based interpretations that may not encompass the full range of contextual information needed for clinical decision-making. If model-based explanations are overly relied upon, physicians may overlook individual patient differences, clinical experience, and other qualitative factors that can be misleading in specific cases. Furthermore, problems of missing, noise, or bias in the data may also affect the accuracy of the results of SHAP analysis.

Therefore, in complex clinical scenarios, it is recommended that clinicians combine the SHAP analysis results with their own professional knowledge and clinical experience so as to make up for the model’s deficiencies in capturing variable interaction effects and avoid misjudgments caused by relying solely on the model interpretation. At the same time, presenting SHAP results through intuitive visualization tools allows decision-makers to better integrate model interpretation with actual clinical situations. During the data acquisition and pre-processing phases, it is also critical to regularly review the data quality to ensure that the data fed into the model is reliable. Combining the above strategies, the clinical team can more effectively leverage the advantages of the SHAP method and achieve more accurate and scientific clinical decision support.

To promote the practical application of the model in the ICU, its integration with clinical workflow needs to be considered. Although the model in this study has good performance and interpretability, it still faces challenges in clinical deployment, such as data availability, computational resource requirements, physician acceptance, and model interpretability. For example, this study used a publicly available database, whereas the electronic medical record systems of different hospitals may have differences in data structure and recording methods, requiring localized adaptation. In addition, clinicians’ trust in the model’s prediction results relies on whether the model has a clear explanation mechanism, such as the SHAP method used in this study. In the future, the model can be integrated into the electronic medical record system to realize automated risk assessment and real-time warning based on patient information, assisting clinicians to make more scientific decisions in high-risk situations, thus enhancing its clinical utility.

Future research could expand this study in several ways. First, more clinical variables, such as medication records and dynamic vital signs, can be introduced to improve the predictive ability and individualization of the model, and attempts can be made to incorporate deep learning methods, such as the attention mechanism, to further enhance the performance and interpretability of the model. Second, given the evolving nature of ICU treatment practices, it is important to explore the model’s adaptability to new ventilation strategies and therapeutic protocols. In addition, this study constructed the model based on two U.S. public databases and has not been validated in external datasets, which is acceptable in initial studies but should still be stated as an important limitation. Future studies could be externally validated on multicenter, cross-regional data to assess the generalizability and clinical portability of the model.

## 5 Conclusion

We developed a model to predict the mortality risk of critically ill ARDS patients in the ICU and validated its performance. To enhance the interpretability of the model, we employed SHAP techniques, which help users identify the key factors that have the greatest impact on ARDS mortality risk. This machine learning model effectively identifies high-risk ARDS patients at an early stage, thereby supporting clinical decision-making, promoting early intervention, and improving patient prognosis.

## Data Availability

The raw data supporting the conclusions of this article will be made available by the authors, without undue reservation.
