# Supplementary material for: An interpretable machine learning model for predicting mortality risk in adult ICU patients with acute respiratory distress syndrome
Source: Front Med (Lausanne). 2025 Apr 25;12:1580345. doi: 10.3389/fmed.2025.1580345 (PMC12061690; doi:10.3389/fmed.2025.1580345)
Supplement: Supplementary file 1 [file Table_1.docx]

Table 1 Mean and median differences between variables before and after imputation

| **Feature** | **Mean** | **95% CI** | **Median** | **95% CI** |
| --- | --- | --- | --- | --- |
| Admission Height | -0.033 | (-0.469, 0.379) | -0.086 | (-2, 2) |
| Admission Weight | 0.015 | (-1.147, 1.146) | 0.023 | (-1.15, 1.2) |
| Heart Rate | 0.081 | (-1.021, 1.156) | 0.058 | (-1, 1) |
| Respiratory Rate | 0.045 | (-0.447, 0.548) | 0.043 | (-0.66, 1) |
| DBP | -0.332 | (-1.227, 0.525) | -0.145 | (-1, 1) |
| SBP | 0.555 | (-0.661, 1.829) | 1.143 | (-1, 3) |
| MBP | 0.166 | (-1.365, 1.593) | 1.462 | (-0.746, 3) |
| Temperature | -0.001 | (-0.044, 0.025) | -0.016 | (-0.076, 0.043) |
| Average Blood Glucose | 0.037 | (-2.579, 2.670) | 0.087 | (-2, 2.5) |
| PaO2 | -0.188 | (-3.665, 3.070) | 1.862 | (-0.002, 4) |
| PaCO2 | -0.110 | (-0.816, 0.581) | 0.073 | (-0.5, 0.7) |
| SpO2 | -0.079 | (-0.315, 0.176) | -0.004 | (-0.077, 0) |
| FiO2 | -0.003 | (-0.013, 0.008) | 0 | (0, 0) |
| PH | -0.000 | (-0.004, 0.004) | -0.001 | (-0.007,0.001) |
| Base Excess | -0.325 | (-0.596, -0.081) | -0.460 | (-0.7, -0.2) |
| Hematocrit | -0.012 | (-0.259, 0.226) | -0.024 | (-0.326, 0.25) |
| Hemoglobin | -0.003 | (-0.089, 0.079) | -0.003 | (-0.1, 0.1) |
| Platelet | 0.268 | (-7.814, 8.055) | 0.354 | (-8.005, 9) |
| WBC | 0.018 | (-0.437, 0.455) | 0.020 | (-0.203, 0.25) |
| Albumin | 0.000 | (-0.023, 0.025) | -0.006 | (-0.05, 0.05) |
| Anion Gap | 0.049 | (-0.126, 0.215) | 0.140 | (-0.4, 0.5) |
| Bicarbonate | 0.067 | (-0.154, 0.284) | 0.084 | (-0.5, 0.5) |
| Total bilirubin | -0.020 | (-0.108, 0.080) | 0.041 | (0.004, 0.05) |
| BUN | 0.010 | (-0.848, 0.807) | -0.024 | (-1, 1) |
| Calcium | 0.003 | (-0.029, 0.033) | 0.000 | (0, 0) |
| Mean Chloride | 0.008 | (-0.238, 0.250) | -0.002 | (-0.5, 0.5) |
| Mean Creatinine | 0.000 | (-0.061, 0.059) | 0.000 | (-0.035, 0.035) |
| Mean Sodium | 0.003 | (-0.199, 0.194) | 0.011 | (-0.5, 0.5) |
| Mean Potassium | 0.000 | (-0.022, 0.022) | 0.000 | (-0.026, 0.05) |
| ALT | 0.062 | (-8.381, 7.951) | 1.301 | (0, 2) |
| ALP | 0.109 | (-4.050, 4.042) | 1.755 | (0, 3.6) |
| AST | -0.403 | (-12.013, 10.832) | 1.612 | (0.098, 3) |
| APACHE | 0.339 | (-0.657, 1.293) | 0.390 | (-1, 2) |
| GCS | 0.065 | (-0.057, 0.200) | -0.175 | (-0.4, 0) |

DBP: Diastolic Blood Pressure; SBP: Systolic Blood Pressure; PaO_2_: Partial Pressure of Oxygen; PaCO_2_: Partial Pressure of Carbon Dioxide; SpO_2_: Oxygen Saturation, Pulse Oximetry; FiO_2_: Fraction of Inspired Oxygen; PH: Hydrogen Ion Concentration, Acidity/Alkalinity; BUN: Blood Urea Nitrogen; ALT: Alanine Aminotransferase; ALP: Alkaline Phosphatase; AST: Aspartate Aminotransferase; APACHE: Acute Physiology and Chronic Health Evaluation; GCS: Glasgow Coma Scale.

Table 2 Features selected by RFECV and importance values from DT.

| **Feature** | **Importance** | **Feature** | **Importance** |
| --- | --- | --- | --- |
| Age | 0.0 | Albumin | 0.055142 |
| Admission Height | 0.0 | Platelet | 0.055914 |
| Admission Weight | 0.0 | Total bilirubin | 0.010702 |
| BMI | 0.0 | BUN | 0.034401 |
| Length of Hospital Stay | 0.285299 | Calcium | 0.010845 |
| ICU Admission Hours | 0.080409 | Mean Chloride | 0.0 |
| Heart Rate | 0.003683 | Mean Creatinine | 0.0 |
| Respiratory Rate | 0.0 | Mean Sodium | 0.0 |
| DBP | 0.0 | Mean Potassium | 0.0 |
| SBP | 0.115125 | ALT | 0.012895 |
| MBP | 0.0 | ALP | 0.017529 |
| Temperature | 0.0 | AST | 0.041712 |
| Average Blood Glucose | 0.0 | APACHE | 0.242200 |
| PaO_2_ | 0.0 | GCS | 0.0 |
| PaCO_2_ | 0.0 | OASIS | 0.0 |
| SpO_2_ | 0.003596 | Charlson | 0.0 |
| FiO_2_ | 0.0 | Diabetes | 0.0 |
| PaO_2_/FiO_2_ | 0.030548 | Kidney Disease | 0.0 |
| PH | 0.0 | Liver Disease | 0.0 |
| Base Excess | 0.0 | Malignant Neoplasms | 0.0 |
| Hematocrit | 0.0 | Myocardial Infarction | 0.0 |
| Hemoglobin | 0.0 | Heart Failure | 0.0 |
| Peripheral Vascular Disease | 0.0 | Cerebrovascular Disease | 0.0 |

BMI: Body Mass Index; DBP: Diastolic Blood Pressure; SBP: Systolic Blood Pressure; MBP: Mean Blood Pressure; PaO_2_: Partial Pressure of Oxygen; PaCO_2_: Partial Pressure of Carbon Dioxide; SpO_2_: Oxygen Saturation, Pulse Oximetry; FiO_2_: Fraction of Inspired Oxygen; PaO_2_/FiO_2_: Ratio of Partial Pressure of Oxygen to Fraction of Inspired Oxygen; PH: Hydrogen Ion Concentration, Acidity/Alkalinity; BUN: Blood Urea Nitrogen; ALT: Alanine Aminotransferase; ALP: Alkaline Phosphatase; AST: Aspartate Aminotransferase; APACHE: Acute Physiology and Chronic Health Evaluation; GCS: Glasgow Coma Scale; OASIS: Outcomes and Assessment Information Set; Charlson: Charlson Comorbidity Index.

Table 3 Features selected by RFECV and importance values from XGBoost.

| **Feature** | **Importance** | **Feature** | **Importance** |
| --- | --- | --- | --- |
| Age | 0.024640 | Platelet | 0.030504 |
| Admission Height | 0.019274 | WBC | 0.018865 |
| Admission Weight | 0.020470 | Albumin | 0.034498 |
| BMI | 0.020618 | Base Excess | 0.019654 |
| Length of Hospital Stay | 0.061903 | Total bilirubin | 0.022956 |
| ICU Admission Hours | 0.049393 | BUN | 0.027434 |
| Heart Rate | 0.017543 | Calcium | 0.021765 |
| Respiratory Rate | 0.016484 | Mean Creatinine | 0.017557 |
| DBP | 0.020349 | Mean Sodium | 0.017249 |
| SBP | 0.037146 | ALT | 0.018625 |
| Temperature | 0.020532 | ALP | 0.018356 |
| PH | 0.019345 | AST | 0.046607 |
| PaO2 | 0.018131 | APACHE | 0.063033 |
| PaCO2 | 0.016303 | GCS | 0.023579 |
| SpO2 | 0.017683 | OASIS | 0.028951 |
| FiO2 | 0.028176 | Charlson | 0.042056 |
| PaO2/FiO2 | 0.022433 | Diabetes | 0.028317 |
| Hematocrit | 0.019326 | Liver Disease | 0.022952 |
| Hemoglobin | 0.015865 | Malignant Neoplasms | 0.031428 |

BMI: Body Mass Index; SBP: Systolic Blood Pressure; PaO_2_: Partial Pressure of Oxygen; PaCO_2_: Partial Pressure of Carbon Dioxide; SpO_2_: Oxygen Saturation, Pulse Oximetry; FiO_2_: Fraction of Inspired Oxygen; PaO_2_/FiO_2_: Ratio of Partial Pressure of Oxygen to Fraction of Inspired Oxygen; pH: Hydrogen Ion Concentration, Acidity/Alkalinity; WBC: White Blood Cell Count; BUN: Blood Urea Nitrogen; ALT: Alanine Aminotransferase; ALP: Alkaline Phosphatase; AST: Aspartate Aminotransferase; APACHE: Acute Physiology and Chronic Health Evaluation; GCS: Glasgow Coma Scale; OASIS: Outcomes and Assessment Information Set; Charlson: Charlson Comorbidity Index.

Table 4 Features selected by RFECV and importance values from RF.

| **Feature** | **Importance** | **Feature** | **Importance** |
| --- | --- | --- | --- |
| Age | 0.041786 | Platelet | 0.058223 |
| BMI | 0.037541 | WBC | 0.037642 |
| Length of Hospital Stay | 0.105376 | Albumin | 0.045650 |
| ICU Admission Hours | 0.059425 | Total bilirubin | 0.037283 |
| Heart Rate | 0.034969 | BUN | 0.039811 |
| DBP | 0.039738 | Mean Creatinine | 0.035544 |
| SBP | 0.056294 | ALT | 0.035638 |
| PaCO_2_ | 0.036872 | ALP | 0.041197 |
| Base Excess | 0.035673 | AST | 0.063126 |
| PaO_2_/FiO_2_ | 0.045409 | APACHE | 0.066563 |
| OASIS | 0.046241 |  |  |

BMI: Body Mass Index; DBP: Diastolic Blood Pressure; SBP: Systolic Blood Pressure; PaCO_2_: Partial Pressure of Carbon Dioxide; PaO_2_/FiO_2_: Ratio of Partial Pressure of Oxygen to Fraction of Inspired Oxygen; WBC: White Blood Cell Count; ALT: Alanine Aminotransferase; ALP: Alkaline Phosphatase; AST: Aspartate Aminotransferase; APACHE: Acute Physiology and Chronic Health Evaluation; OASIS: Outcomes and Assessment Information Set.

Table 5 Features selected by RFECV and importance values from GBDT.

| **Feature** | **Importance** | **Feature** | **Importance** |
| --- | --- | --- | --- |
| Age | 0.028526 | Platelet | 0.056704 |
| Admission Weight | 0.012082 | Albumin | 0.048187 |
| Length of Hospital Stay | 0.248966 | Total bilirubin | 0.016771 |
| ICU Admission Hours | 0.131708 | BUN | 0.020057 |
| DBP | 0.020740 | ALT | 0.011360 |
| SBP | 0.053635 | ALP | 0.017536 |
| Base Excess | 0.013555 | AST | 0.075457 |
| WBC | 0.010916 | APACHE | 0.097435 |
| Calcium | 0.012963 | OASIS | 0.029601 |
| FiO_2_ | 0.011340 | Charlson | 0.026834 |
| PaO_2_/FiO_2_ | 0.029547 | BMI | 0.010871 |
| Heart Rate | 0.015209 |  |  |

DBP: Diastolic Blood Pressure; SBP: Systolic Blood Pressure; FiO_2_: Fraction of Inspired Oxygen; PaO_2_/FiO_2_: Ratio of Partial Pressure of Oxygen to Fraction of Inspired Oxygen; BUN: Blood Urea Nitrogen; ALT: Alanine Aminotransferase; ALP: Alkaline Phosphatase; AST: Aspartate Aminotransferase; APACHE: Acute Physiology and Chronic Health Evaluation; OASIS: Outcomes and Assessment Information Set; Charlson: Charlson Comorbidity Index; BMI: Body Mass Index; Charlson: Charlson Comorbidity Index.

Table 6 Features selected by RFECV and importance values from AdaBoost.

| **Feature** | **Importance** | **Feature** | **Importance** |
| --- | --- | --- | --- |
| Age | 0.03 | WBC | 0.02 |
| Admission Weight | 0.01 | Albumin | 0.05 |
| Admission Height | 0.03 | BMI | 0.01 |
| Length of Hospital Stay | 0.1 | Bicarbonate | 0.01 |
| ICU Admission Hours | 0.07 | Total bilirubin | 0.02 |
| Heart Rate | 0.02 | BUN | 0.03 |
| Respiratory Rate | 0.02 | Calcium | 0.04 |
| DBP | 0.03 | Mean Chloride | 0.01 |
| SBP | 0.04 | Mean Creatinine | 0.02 |
| Temperature | 0.02 | Mean Sodium | 0.02 |
| AIDS | 0.01 | Mean Potassium | 0.02 |
| PaO_2_ | 0.01 | ALT | 0.02 |
| PaCO_2_ | 0.0 | PH | 0.0 |
| SpO_2_ | 0.0 | ALP | 0.02 |
| FiO_2_ | 0.01 | AST | 0.05 |
| PaO_2_/FiO_2_ | 0.03 | APACHE | 0.06 |
| Base Excess | 0.02 | GCS | 0.01 |
| Hematocrit | 0.05 | OASIS | 0.02 |
| Anion Gap | 0.01 | Charlson | 0.02 |
| Platelet | 0.03 | Diabetes | 0.01 |
| Peripheral Vascular Disease | 0.0 |  |  |

DBP: Diastolic Blood Pressure; SBP: Systolic Blood Pressure; PaO_2_: Partial Pressure of Oxygen; PaCO_2_: Partial Pressure of Carbon Dioxide; SpO_2_: Oxygen Saturation, Pulse Oximetry; FiO_2_: Fraction of Inspired Oxygen; PaO_2_/FiO_2_: Ratio of Partial Pressure of Oxygen to Fraction of Inspired Oxygen; BMI: Body Mass Index; WBC: White Blood Cell Count; BUN: Blood Urea Nitrogen; pH: Hydrogen Ion Concentration, Acidity/Alkalinity; ALT: Alanine Aminotransferase; ALP: Alkaline Phosphatase; AST: Aspartate Aminotransferase; APACHE: Acute Physiology and Chronic Health Evaluation; GCS: Glasgow Coma Scale; OASIS: Outcomes and Assessment Information Set; Charlson: Charlson Comorbidity Index; AIDS: Acquired Immunodeficiency Syndrome;

Table 7 Features selected by RFECV and importance values from LightGBM.

| **Feature** | **Importance** | **Feature** | **Importance** |
| --- | --- | --- | --- |
| Age | 161 | Platelet | 191 |
| Admission Height | 126 | WBC | 160 |
| Admission Weight | 127 | Albumin | 168 |
| BMI | 126 | Anion Gap | 117 |
| Length of Hospital Stay | 473 | Bicarbonate | 96 |
| ICU Admission Hours | 347 | Total bilirubin | 132 |
| Heart Rate | 171 | BUN | 135 |
| Respiratory Rate | 126 | Calcium | 112 |
| DBP | 157 | Mean Chloride | 88 |
| SBP | 165 | Mean Creatinine | 144 |
| MBP | 135 | Mean Sodium | 85 |
| Temperature | 110 | Mean Potassium | 136 |
| Average Blood Glucose | 174 | ALT | 134 |
| PaO_2_ | 118 | ALP | 180 |
| PaCO_2_ | 120 | AST | 179 |
| SpO_2_ | 118 | APACHE | 179 |
| FiO_2_ | 77 | GCS | 74 |
| PaO_2_/FiO_2_ | 175 | OASIS | 148 |
| PH | 114 | Charlson | 66 |
| Base Excess | 150 | Hemoglobin | 104 |
| Hematocrit | 102 |  |  |

BMI: Body Mass Index; DBP: Diastolic Blood Pressure; SBP: Systolic Blood Pressure; MBP: Mean Blood Pressure; PaO_2_: Partial Pressure of Oxygen; PaCO_2_: Partial Pressure of Carbon Dioxide; SpO_2_: Oxygen Saturation, Pulse Oximetry; FiO_2_: Fraction of Inspired Oxygen; PaO_2_/FiO_2_: Ratio of Partial Pressure of Oxygen to Fraction of Inspired Oxygen; pH: Hydrogen Ion Concentration, Acidity/Alkalinity; WBC: White Blood Cell Count; BUN: Blood Urea Nitrogen; ALT: Alanine Aminotransferase; ALP: Alkaline Phosphatase; AST: Aspartate Aminotransferase; APACHE: Acute Physiology and Chronic Health Evaluation; GCS: Glasgow Coma Scale; OASIS: Outcomes and Assessment Information Set; Charlson: Charlson Comorbidity Index.

Table 8 Hyperparameter combinations for DT after Bayesian Optimization

| **Hyperparameter** | **Search Interval** | **Value** |
| --- | --- | --- |
| criterion | ['gini', 'entropy'] | entropy |
| max_depth | (3, 15) | 5 |
| min_samples_leaf | (2, 50) | 10 |
| min_samples_split | (1, 10) | 26 |

Table 9 Hyperparameter combinations for XGBoost after Bayesian Optimization

| **Hyperparameter** | **Search Interval** | **Value** |
| --- | --- | --- |
| colsample_bytree | (0.3, 1.0) | 0.5806440809429173 |
| gamma | (0, 10) | 0 |
| learning_rate | (0.01, 0.5) | 0.01 |
| max_depth | (3, 20) | 12 |
| min_child_weight | (1, 15) | 4 |
| n_estimators | (50, 1000) | 715 |
| reg_alpha | (0, 5) | 0 |
| reg_lambda | (0.1, 10) | 0.1 |
| subsample | (0.3, 1.0) | 0.8835787825477399 |
| scale_pos_weight | (1, 8) | 3.337362556680439 |

Table 10 Hyperparameter combinations for RF after Bayesian Optimization

| **Hyperparameter** | **Search Interval** | **Value** |
| --- | --- | --- |
| max_depth | (3, 15) | 15 |
| max_features | (0.1, 1.0) | 1.0 |
| min_samples_leaf | (1, 20) | 10 |
| min_samples_split | (2, 20) | 2 |
| n_estimators | (50, 800) | 800 |

Table 11 Hyperparameter combinations for GBDT after Bayesian Optimization

| **Hyperparameter** | **Search Interval** | **Value** |
| --- | --- | --- |
| learning_rate | (0.01, 0.3) | 0.01 |
| max_depth | (3, 15) | 13 |
| min_samples_leaf | (1, 10) | 10 |
| min_samples_split | (2, 20) | 7 |
| n_estimators | (50, 800) | 733 |
| subsample | (0.5, 1.0) | 0.6376982665531987 |

Table 12 Hyperparameter combinations for Ensemble learning after Bayesian Optimization

| **Hyperparameter** | **Search Interval** | **Value** |
| --- | --- | --- |
| gb__learning_rate | (0.001, 0.1) | 0.07789444974544028 |
| gb__n_estimators | (50, 800) | 428 |
| rf__max_depth | (3, 20) | 20 |
| rf__n_estimators | (50, 800) | 800 |
| svm__C | (0.01, 10) | 0.01 |
| svm__gamma | (0.001, 1) | 0.001 |
| xgb__max_depth | (3, 20) | 16 |
| xgb__n_estimators | (50, 800) | 50 |

Table 13 Hyperparameter combinations for AdaBoost after Bayesian Optimization

| **Hyperparameter** | **Search Interval** | **Value** |
| --- | --- | --- |
| learning_rate | (0.01, 1.0) | 0.3736861858218207 |
| n_estimators | (50, 800) | 800 |

Table 14 Hyperparameter combinations for LightGBM after Bayesian Optimization

| **Hyperparameter** | **Search Interval** | **Value** |
| --- | --- | --- |
| colsample_bytree | (0.5, 1.0) | 0.5 |
| learning_rate | (0.01, 0.3) | 0.01834567330758717 |
| max_depth | (3, 15) | 10 |
| min_child_samples | (10, 100) | 100 |
| n_estimators | (50, 800) | 399 |
| num_leaves | (31, 255) | 31 |
| reg_alpha | (0.001, 1.0) | 0.001 |
| reg_lambda | (0.001, 1.0) | 1.0 |
| subsample | (0.5, 1.0) | 1.0 |

Table 15 DeLong test tesults with Holm-Bonferroni correction for AUC-ROC comparison between XGBoost and other models

| Model | P-value (Unadjusted) | P-value (Holm-Bonferroni Corrected) |
| --- | --- | --- |
| DT | 4.272077e-16 | 3.417662e-15 |
| RF | 8.575067e-06 | 4.287534e-05 |
| GBDT | 1.005085e-01 | 3.015254e-01 |
| Ensemble | 4.800794e-04 | 1.920317e-03 |
| Adaboost | 8.463088e-10 | 5.077853e-09 |
| LightGBM | 1.500622e-01 | 3.015254e-01 |
| BPNN | 9.374124e-13 | 6.561887e-12 |

Table 16 Subgroup analysis of XGBoost for predicting risk of mortality in ARDS patients

| Category | Subgroup | AUC | 95% CI |
| --- | --- | --- | --- |
| Age | 18-39 | 0.977 | (0.930, 1) |
| Age | 40-64 | 0.880 | (0.830, 0.924) |
| Age | ≥65 | 0.872 | (0.838, 0.901) |
| Sex | Male | 0.895 | (0.863, 0.922) |
| Sex | Female | 0.876 | (0.837, 0.909) |
| Liver Disease | Without Condition | 0.883 | (0.856, 0.908) |
| Liver Disease | With Condition | 0.881 | (0.797, 0.951) |
| Renal Disease | Without Condition | 0.887 | (0.860, 0.911) |
| Renal Disease | With Condition | 0.886 | (0.823, 0.940) |
| Chronic Pulmonary Disease | Without Condition | 0.885 | (0.855, 0.912) |
| Chronic Pulmonary Disease | With Condition | 0.892 | (0.848, 0.932) |


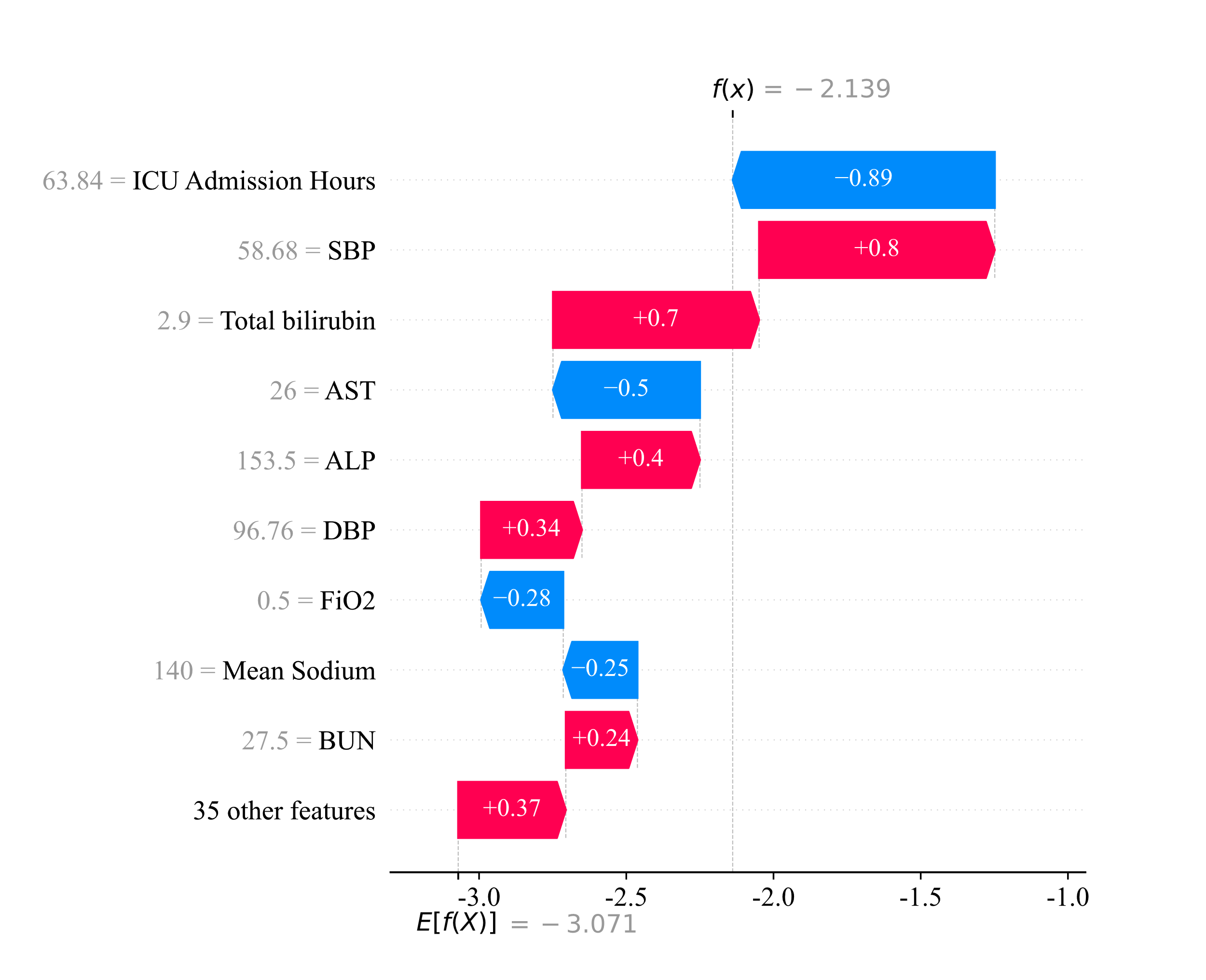


Fig. 1. Waterfall plot of XGBoost predicting ARDS mortality risk (non-surviving samples). SBP: Systolic Blood Pressure; AST: Aspartate Aminotransferase; ALP: Alkaline Phosphatase; DBP: Diastolic Blood Pressure; FiO_2_: Fraction of Inspired Oxygen; BUN: Blood Urea Nitrogen.


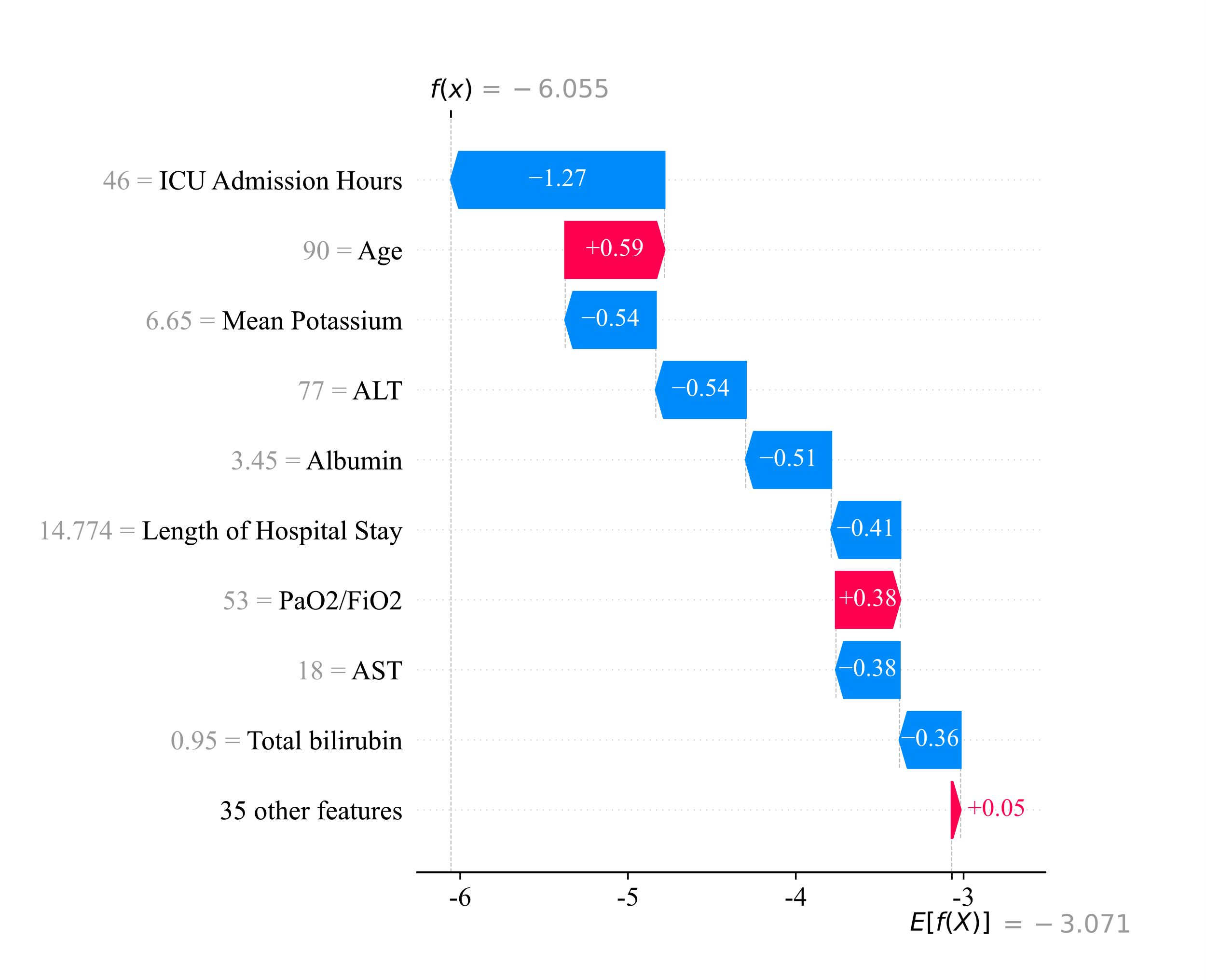


Fig. 2. Waterfall plot of XGBoost predicting ARDS mortality risk (surviving samples). ALT: Alanine Aminotransferase; AST: Aspartate Aminotransferase; PaO_2_/FiO_2_: Ratio of Partial Pressure of Oxygen to Fraction of Inspired Oxygen.
